# Potential Protective Role of Diabetes Mellitus on Aortic Aneurysms

**DOI:** 10.1055/a-2624-0594

**Published:** 2025-08-04

**Authors:** Eliza Epstein, John A. Elefteriades

**Affiliations:** 1 Yale University School of Medicine, New Haven, Connecticut

**Keywords:** abdominal aortic aneurysm, thoracic aortic aneurysm, diabetes mellitus

## Abstract

**Background:**

Prior research provided evidence that diabetes mellitus (DM) may convey protection to patients with abdominal aortic aneurysm (AAA) and/or thoracic aortic aneurysm (TAA).

**Methods:**

We sought recent publications that support or elaborate on this concept using PubMed and Cochrane, searching for publications that combine the search terms “aortic aneurysm” and “diabetes mellitus.” We collate and summarize evidence from the literature on this topic.

**Results:**

We examined pertinent data on AAA, TAA, and aortic aneurysms in general (AA). Patients with DM have lower risk of developing AAA and a lower rate of growth of AAA. Patients with DM have a lower risk of mortality following hospitalizations for AA. That said, however, patients with DM who undergo AAA repair show higher risk of mortality. Patients with DM have lower aneurysm diameter and lower homocysteine and D-dimer levels. Research is emerging regarding a possible genetic explanation: the gene *PSMD12* may play a role in the connection between AAA and DM. Patients with AAA taking diabetic medication metformin show reduced rate of growth of AAA as well as decreased mortality and complications. In TAA, however, no statistically significant differences in mortality or complications are consistently found. We find positive evidence to support the concept that diabetes does confer protection from AAA rupture. Current data does confirm significant protective effect for TAA.

**Conclusion:**

We confirm that metformin does exert protective properties. Diabetic protection against AAA may be mediated via Laplace's Law, as diabetic aortas have thicker walls, thus decreasing wall tension.

Previous research has suggested that diabetes mellitus (DM) may play a protective role in aortic aneurysm (AA) disease. 1 This review compiles studies published between 2018 and 2024 that explore this relationship.

DM has proven to have many potential negative impacts on the heart. Individuals with DM are significantly more likely to develop cardiovascular disease (CVD) and are 1.7 times more likely to die of CVD than those in a general population. 2 However, in contrast to the substantial general cardiovascular risks posed by DM, multiple studies (see below) have suggested that DM exerts a protective impact on AAs. In the operating room, we routinely find the aorta to be thicker in patients with DM, thereby decreasing the risk of growth of AA and aortic dissections. Laplace's Law states that T = P × r (where P is pressure, r is radius, and T is wall thickness). Thus, in simple terms, a thicker aorta “feels” less wall tension at a given pressure and radius. This fundamental scientific relationship may represent a method of action for diabetic protection, as diabetic aortas are thicker than nondiabetic. Primary studies have aimed to support the diabetic hypothesis but left substantial gaps in terms of potential methods and stages of protection in the clinical course of AA disease.

This paper explores recent studies (from 2018 to present) to understand if, when, and how a patient with AAs might benefit clinically from a diabetic state. In the initial sections of this review, we provide background information on diabetes and AA. Next, in the literature review, relevant studies are categorized into two sections: one on AAA and one on TAA. We cover multiple topics: (1) potential underlying biological mechanisms of diabetic protection, (2) long-term risks of aneurysm disease (with and without diabetes), (3) relevant genetics, and (4) a potential role of diabetic medications. We aim to provide up-to-date clarification regarding the putative protective role of diabetes in AA disease via these efforts.

## Background

“Aortic aneurysm” is defined as a dilated area of the aorta (≥4.0 cm for the ascending aorta or ≥3 cm for the descending or abdominal aorta).

AAs in the ascending aorta differ fundamentally from those in the descending/abdominal region. Abdominal aortic aneurysms (AAA) have been shown to involve inflammation of the aortic wall, smooth muscle cell (SMC) apoptosis, and resultant oxidative stress in the tissues, which further activates proteases, leading to degradation of the extracellular matrix as well further activation of inflammatory pathways. Since original studies in the 1960s and 1970s by Tilson and Seashore at Yale, 3 AAA was shown to be familial, with clear genetic predisposition. In the late 1990s, Biddinger et al 4 and our Yale group 5 independently demonstrated clear familial patterns in TAA. TAA is especially genetic in origin, compared with AAA.

Syndromic diseases, in which patients exhibit extra-aortic manifestations in addition to AAs, were demonstrated early (as early 1896). These include Marfan's, Ehlers–Danlos, and Loeys–Dietz syndromes. Subsequently, 72 separate genes have been associated to some degree with TAA. 6 Studies have shown that TAA can often stem from disturbances related to (1) extracellular matrix degeneration, (2) SMC disruption, (3) abnormal transforming growth factor beta regulation, and (4) excess inflammation. The matrix metalloproteinases (MMPs) have been shown to play a major role in degrading the connective tissue of the aorta; thus, increased levels of MMP can contribute to aneurysm formation. 7

Multiple potential mechanisms have been proposed for putative protective effects of diabetes on AAs. Studies have shown that a decrease in wall stress, reduced levels of destructive MMPs, and increased advanced glycation end products may contribute to the amelioration of aneurysm disease. 8 Others have suggested that it is not the disease itself, which is the protector but rather certain medications used to treat the diabetes itself that provide the protective effect.

## Methods

This literature review includes publications within PubMed and Cochrane. The search is focused on papers combining “aortic aneurysm” and “diabetes mellitus” published during or after 2018.

## Results

### General Findings

Given prior emerging evidence of diabetic protection in AAA, more recent studies have endeavored to confirm and understand the mechanisms of this protective relationship ( Table 1 ).

**Table 1:** Natural history of abdominal aortic aneurysm: impact of diabetes

	Title	Key findings: impact(s) of diabetes
Larsson et al (2018) 9	Type 1 and Type 2 diabetes mellitus and incidence of seven cardiovascular diseases	Decreased risk of AAA Increased risk of myocardial infarction, ischemic stroke, heart failure, aortic valve stenosis
Nordness et al (2021) 10	The effect of diabetes on abdominal aortic aneurysm growth over 2 years	35% reduction in growth rate of AAA
Betancourt-Garcia et al (2019) 11	Diabetes and its effect on abdominal aortic aneurysm growth rate in Hispanic patients	65% reduction in monthly expansion of AAA
Ning et al (2020) 12	Diabetes, its duration, and the long-term risk of abdominal aortic aneurysms: the Atherosclerosis Risk in Communities (ARIC) study	30–50% reduction in risk of suffering from AAA Smaller aortic diameters among long-duration diabetics
Avdic et al (2018) 13	Reduced long-term risk of aortic aneurysm and aortic dissection among individuals with Type 2 diabetes mellitus: a nationwide observational study	12% reduction in risk of death following hospitalization for AA No difference in risk of death after aortic dissection
Koba et al (2023) 14	Risk factors for mortality from aortic aneurysm and dissection: results from a 26-year follow-up of a community-based population	Lower multivariable hazard ratio (0.50) for mortality from aortic diseases among diabetics
Li et al (2021) 15	Diabetes mellitus lowers the risk of aortic dissection: a systematic review and meta-analysis	Decreased risk of thoracic aortic dissection among diabetics

Abbreviations: AA, aortic aneurysm; AAA, abdominal aortic aneurysm.

A more generally directed paper 9 analyzed the relationships between DM and CVDs in 71,483 Swedish patients. Supporting previous studies, the authors found that Type 2 diabetes lowers the risk of AAA (hazard ratio [HR]: 0.57; 95% confidence interval [CI]: 0.40–0.82) while, as expected, increasing the risk of myocardial infarction, ischemic stroke, and heart failure.

Nordness et al 10 analyzed the relationship between diabetes and aneurysm growth rate among 250 patients: 56 with diabetes and 194 without. There was no statistically significant difference between baseline maximum diameters of those with and without diabetes, but there was a significant difference in median growth rate: 0.12 cm/year for those with diabetes and 0.19 cm/year for those without (a 35% reduction in median growth rates). Additionally, Betancourt et al 11 confirmed the reduced growth rate of AAA among 201 patients (43.5% with DM) in a Hispanic population. The average monthly growth rate of AAA was 0.07 mm for those with DM and 0.21 mm for those without (a 65% reduction). Thus, two studies support the conclusion that diabetics with AAA experience less growth over the years—a substantial evidence of “benefit” from diabetes.

Ning et al 12 examined the long-term impacts of diabetes on the aorta. Long-duration diabetics exhibited smaller aortic diameters compared with those without diabetes. Those with long-term diabetes had a significantly lower risk of suffering from AAA: a 30 to 50% reduction in risk of AAA 8 years after being diagnosed with diabetes.

Avdic et al 13 examined the impact of diabetes on mortality after AAA or TAA diagnosis, using the Swedish National Diabetes Registry. Patients with Type II DM benefited from a 12% relative risk (RR) reduction of death following hospitalization for AA. However, the authors found no difference in the risk of death at 2 years after aortic dissection. In an important 26-year study, Koba et al 14 found a lower multivariable HR for mortality among AAA patients with diabetes compared with those without (HR: 0.50 [0.28–0.89]) Furthermore, a focused, highly specific systematic review, a meta-analysis 15 showed that diabetes significantly decreased the risk of aortic dissection among patients with diabetes.

Despite different viewpoints, approaches, endpoints, and durations these recently emerging studies all found that patients with diabetes exhibited advantages, including lower likelihood of developing AA over time, reduced growth rate of AAA once it has developed, decreased risk of death after hospitalization for AAA, and lower rate of aortic dissection—all beneficial effects of diabetes on AA. The studies discussed in this section revolve predominantly, although not exclusively, around abdominal rather than thoracic AAs.

### Impact of Diabetes following Aortic Aneurysm Resection

Given the protective effects that DM exhibits on AAs, recent research has examined the effect that DM has on outcomes after AA resection ( Table 2 ).

**Table 2:** Influence of diabetes on perioperative outcomes

Zarrouk et al (2019) 16	Long-term survival and cardiovascular morbidity after elective open aortic aneurysm repair in patients with and without Type 2 diabetes: a nationwide propensity-adjusted analysis	No statistically significant difference in mortality DM/no DM Higher rate of perioperative cardiac events in DM group
Raffort et al (2021) 17	Nationwide study in France investigating the impact of diabetes on mortality in patients undergoing abdominal aortic aneurysm repair	Higher rate of perioperative cardiac events in DM group Decreased survival in DM group Lower rates of DM in ruptured compared with unruptured AAA
Takahara et al (2022) 18	Clinical features and prognosis of patients with and without diabetes mellitus undergoing endovascular aortic aneurysm repair	Higher mortality for patients with DM following AAA repair Lower rates of DM in ruptured AAA as compared with unruptured AAA
Taimour et al (2019) 19	Nationwide comparison of long-term survival and cardiovascular morbidity after acute aortic aneurysm repair in patients with and without Type 2 diabetes	Lower rates of general mortality and cardiovascular mortality following acute AAA repair in DM group DM exerts positive perioperative effects on aortic wall No significant difference in cardiovascular events, acute myocardial infarction, and stroke
Taimour et al (2019) 20	Survival, cardiovascular morbidity and reinterventions after elective endovascular aortic aneurysm repair in patients with and without diabetes: a nationwide propensity-adjusted analysis	Lower need for reintervention after endovascular repair in DM Higher rates of acute MI

Abbreviations: AAA, abdominal aortic aneurysm; DM, diabetes mellitus; MI, myocardial infarction.

Zarrouk et al 16 performed a nationwide observational cohort study comparing 397 patients with Type 2 DM who underwent elective open AAA resection to 1,709 patients without diabetes who underwent the same surgical procedure, using data from the Swedish Registry and the Swedish National Diabetes Register. Median follow-up was 4.51 years for patients with diabetes and 4.59 years for those without. The authors found no statistically significant difference in mortality between the two groups. However, patients with diabetes showed significantly higher rates of acute myocardial infarction and major adverse cardiovascular events during follow-up. Further, a 10-year retrospective study 17 using the French National Electronic health data from 2010 to 2019 found higher mortality for patients with diabetes following AAA repair. A study in Japan 18 also found that patients with diabetes undergoing endovascular aortic repair had higher risks of mortality and cardiovascular events, with lower long-term survival. As in other studies, they also found much lower rates of diabetes in the ruptured AAA patients than those without rupture (2.5% compared with 4.0% for Type 1 DM, and 14.8% compared with 20.9% for Type 2 DM).

Alternatively, a Swedish study 19 (mean follow-up 3.8 years) found that those with Type 2 diabetes had lower rates of general mortality and cardiovascular mortality following acute AAA repair (1,375 open and 850 endovascular) than those without DM (RR: 0.75; 95% CI: 0.59–0.95; *p* = 0.016), but there was no significant difference in cardiovascular events, acute myocardial infarction, and stroke between the two groups. The conclusion drawn in this study was that diabetes has positive effects on the aortic wall, decreasing mortality during and following surgery. Similarly, a Nationwide observational cohort study of patients in Sweden 20 found that those with diabetes showed a lower need for reintervention following endovascular aneurysm repair (EVAR), but higher rates of acute myocardial infarctions during follow-up. There was no significant difference in total mortality, cardiovascular mortality, or stroke during follow-up.

In sum, some of these studies, but not all, found surgery technically safer in diabetes (due to a stronger aortic wall). However, most studies found increased perioperative susceptibility to primary cardiac events (such as myocardial infarction).

### Potential Mechanisms of Aortic Protection

While many studies over the years have indicated that DM exerts a protective effect against aortic events in AA disease, the mechanisms for such benefits have been unclear. Some authorities have postulated that the medications used to treat DM may underlie the observed benefits ( Table 3 ).

**Table 3:** Potential mechanisms of aortic protection

Xu et al (2022) 21	Influencing factors on diabetes mellitus on abdominal aortic aneurysm diameter and biochemical parameters	Lower aneurysm diameter and homocysteine and D-dimer levels among those with diabetes
Morris et al (2019) 22	Opposite associations of aortic aneurysm with blood glucose and with diabetes mellitus	Higher blood glucose manifested as higher rates of AAA (among patients without a diagnosis of diabetes) (contrary to “protection” theory

Abbreviation: AAA, abdominal aortic aneurysm.

A retrospective analysis in Peking 21 examined this question. Patients were separated into two groups: 85 patients with small and medium AAA and 72 with large AAAs. Those with diabetes had higher rates of hypertension (χ ^2^ = 8.147, *p* = 0.004) but smaller aneurysm diameter and lower homocysteine and D-dimer levels than those without diabetes ( *t* = − 3.148, *p* = 0.002; *U* = − 1.503, *p* = 0.013; *U* = − 3.002, *p* = 0.003). The researchers speculate that smaller aortic size (∼lower growth rate) in AAA in patients with diabetes may be consequences of associated treatments with drugs such as metformin, mediated through lower inflammatory indices and D-dimer levels.

In a separate attempt to understand diabetic protection, another study 22 addressed the hypothesis that the relationship between diabetes and AAA was mediated by high levels of blood glucose. The results ran contrary to expectations, showing an opposite effect. Among patients without a diagnosis of DM, higher blood glucose patients manifested higher rates of AAA. For every 2 mmol/L of blood glucose elevation, there was a higher likelihood of AA, with an odds ratio of 1.36. The researchers concluded that it is unlikely that hyperglycemia exerts the protective effect. Rather, they speculate the benefit may result from medical treatments for diabetes that restrain aneurysm growth and dissection.

### Possible Role of Genetics

Given that genetics play a significant role in both Type 2 diabetes and in AAA, recent studies have examined whether the relationship between the two pathologies can be explained through genetic mechanisms ( Table 4 ). One study 24 aimed to identify candidate genes that explain the connection between AAA and Type 2 diabetes. Using the Biomedical Discovery Support System, the investigators screened 86 candidate intermediate molecules that are related to both DM and AA. Through further investigation, it was found that the gene PSMD12 (proteasome 26S subunit, non-ATPase 12) may be associated with the connection between AAA and DM due to its high expression in the aorta as well as pancreatic tissue. If the diabetes—AA connection was to trace-back to a gene, this could help design future treatments.

**Table 4:** Potential role of genetics

Zu et al (2013) 23	Identify candidate genes in the interaction between abdominal aortic aneurysm and Type 2 diabetes mellitus by using a biomedical discovery support system	PSMD12 (proteasome 26S subunit, non-ATPase 12) highly expressed in both aorta and pancreas
Morris et al (2022) 24	Genetic predisposition to diabetes and abdominal aortic aneurysm: a two stage Mendelian randomization study	Utilizing mendelian randomization, no decreased risk of AAA found associated with genetic predisposition to diabetes

Abbreviation: AAA, abdominal aortic aneurysm.

Looking from a different perspective, a meta-analysis 25 was performed to examine whether genetic predisposition to diabetes was shown to protect against AAA using 4,972 cases with 99,585 controls. The researchers used Diabetes Meta-Analysis of Trans-Ethnic association studies (DIAMANTE) and the International Aneurysm Consortium database (including six genome-wide AAA studies) to compare single nucleotide polymorphisms, in search of an association between the single nucleotide polymorphisms involved in DM and AAA. Utilizing Mendelian randomization, it was found that there was not a decreased risk of AAA associated with genetic predisposition to diabetes.

### Potential Role of Diabetes Mellitus Medications

Previous work has shown that diabetes medications have beneficial effects on the aorta. Accordingly, some authorities argue that the protective impacts observed are the result of drug effects and not the disease itself. Recent studies have analyzed the impacts of both metformin and SGLT-2 inhibitors on AAs ( Table 5 ).

**Table 5:** Role of medications (metformin) in aortic protection in diabetic patients

Itoga et al (2019) 25	Metformin prescription status and abdominal aortic aneurysm disease progression in the U.S. Veteran population	20% decrease in unadjusted mean growth rate of AAA for patients taking metformin
Turowicz et al (2021) 26	Association of metformin and abdominal aortic aneurysm repair outcomes	Significant decrease in rates of mortality and complications from AA repair among patients taking metformin
Sutton et al (2020) 27	Association between metformin and abdominal aortic aneurysm in diabetic and non-diabetic US veterans	Decreased need for surgery and risk of death within the first 10 y of AAA diagnosis among patients taking metformin
Ortega et al (2019) 28	SGLT-2 inhibition reduces angiotensin II-induced dissecting abdominal aortic aneurysm in ApoE knockout mice	SGLT-2 inhibitor decreased the degradation of elastin, lowered the formation of neo-vessels and reduced the number of macrophages invading the AAA lesions in dissection-induced mice

Abbreviations: AA, aortic aneurysm; AAA, abdominal aortic aneurysm.

Itoga et al 25 analyzed enlargement of AAAs in relation to metformin usage among 13, 834 patients (who had AAA along with a diagnosis of DM) within the Veterans Affairs Health Care System over a 10-year period. Those prescribed metformin manifested a 20% decrease in unadjusted mean rate of AAA growth. The general conclusion reached was that metformin itself correlated with decreased AAA enlargement in this large Veteran population with both DM and AAA disease. Similarly, Turowicz et al 26 found that metformin significantly reduced morality ( *p* = 0.019) and complications ( *p* = 0.032) of AAA as compared with those with AAA and diabetes on other (nonmetformin) treatments. These studies support the viewpoint that diabetes itself does not impact the aorta but rather the drug treatment itself (metformin) causes the beneficial physiological alterations.

A notable study 27 compared the need for surgery as well as death rates between those with and without diabetes and with or without metformin. This was a retrospective cohort study (2000–2019) looking at three transition phases among veterans (AAA surgery, death, and death after AAA surgery). Individuals with diabetes, with or without metformin, manifested a lower risk of requiring surgery than those without diabetes. Patients with diabetes taking metformin were shown to have the lowest risk of all-cause death, compared with individuals without diabetes, within the first 10 years of AAA diagnosis. That said, after 10 years, that risk reversed: those on metformin with diabetes manifested a higher risk of death than those without diabetes. Patients with diabetes not taking metformin had a higher risk of death before and after surgery. Among their studied populations, individuals with diabetes who are not taking diabetic medications were shown to have the highest risk of death before and after surgery. Overall, those with diabetes taking metformin were found to have lower need for surgery and lower risk of death within the first 10 years of AAA diagnosis.

In addition to the impacts of metformin, another class of medications utilized for diabetes are the SGLT-2 inhibitors. Ortega et al 28 analyzed the effects of chronic oral treatment with empagliflozin (an SGLT-2 inhibitor) on dissecting AAAs in mice. In angiotensin II-induced dissections, empagliflozin decreased the degradation of elastin, lowered the formation of neo-vessels, and reduced the number of macrophages invading the AAA lesion. SGLT-2 inhibitor (empagliflozin) reduced AAA formation (in this mouse study), indicating that SGLT-2 inhibitors could be considered for use in humans to prevent AAA growth.

These studies, whose general thrust is that metformin is protective, raise the provocative question of whether individuals who do not have diabetes yet are diagnosed with AAs should be prescribed these medications originally intended for diabetes, given their apparent substantial protective impacts on AA—reducing AA growth and reducing mortality.

### Thoracic Aortic Aneurysm

While there are fewer recent studies analyzing the association between DM and thoracic aortic aneurysms (TAA), some new developments are noteworthy (See Table 6 ).

**Table 6:** Diabetes and thoracic aortic aneurysm

Summers et al (2023) 29	The association between diabetes mellitus and its management with outcomes following endovascular repair for descending thoracic aortic aneurysm	No significant difference in perioperative mortality or in-hospital complications between those with DM on either insulin or noninsulin medications and those without DM Increase in perioperative and 5-year mortality among diet-controlled DM patients
Gambardella et al (2023) 30	Diabetes mellitus is an independent predictor of spinal cord injury after descending thoracic and thoracoabdominal aneurysm repair: maximum likelihood conditional regression in a propensity-score matched cohort	Patients with DM undergoing TAA repairs had higher rates of spinal cord injuries (6.5% compared with 1.6%) and high rates of operative mortality (14.1% compared with 6%)
Wu et al (2023) 31	Fate of the unoperated ascending thoracic aneurysm: three-decade experience from the aortic institute at Yale University	Decreased hazard ratio of 0.68 for experiencing an adverse aortic event (rupture, dissection, death) for those with DM. However, the *p* -value of 0.520 was not statistically significant
Kalogerakos et al (2021) 32	Root dilatation is more malignant than ascending aortic dilation	No clinically or statistically significant impact was found

Abbreviations: TAA, thoracic aortic aneurysm; DM, diabetes mellitus.

Following similar questions about the impact of diabetes on surgical repair for AAA, two recent studies examined outcomes for TAA repair. Summers et. al 29 studied the relation between having diabetes and outcomes after thoracic EVAR for descending thoracic aortic aneurysm (DTAA) among 2,673 patients, 472 of whom had a confirmed diagnosis of diabetes. Among the group with diabetes, 25% were diet-controlled, 54% were treated with noninsulin medications (usually metformin), and 21% were undergoing insulin therapy. Those who were diet-controlled or receiving insulin management (not metformin) showed higher rates of spontaneous pretreatment rupture than those on noninsulin medications (and those without diabetes). There was no significant difference in perioperative mortality or in-hospital complications between those with diabetes on either insulin or noninsulin medications and those without diabetes. Those with solely dietary management showed higher perioperative mortality as well as a higher 5-year mortality compared with the other group.

Gambardella et al 30 conducted a study that took a nuanced approach. They found that diabetes independently predicted spinal cord injuries among patients undergoing thoracic and thoracoabdominal aneurysm repairs. Analyzing 934 patients who had undergone descending thoracic and thoracoabdominal aneurysm repairs, they found that patients with patients with diabetes had higher rates of spinal cord injury (6.5% compared with 1.6%) and higher rates of operative mortality (14.1% compared with 6%). Further, those with diabetes showed reduced survival at 1 (70.2 vs. 86.2%), 5 (50.4 vs. 67.5%), and 10 years (31.7 vs. 36.7%) compared with the control group. These results serve as a reminder that despite the protective effects of diabetes on AA growth, diabetes leads to many cardiovascular complications that seemingly override the protective mechanisms in terms of overall outcomes.

While research is still limited, Wu et al 31 examined the impact of diabetes on the natural history of ascending thoracic aneurysms. Of the 964 patients analyzed, 79 (8.2%) had diabetes. Interestingly, the authors found a decreased HR of 0.68 for experiencing an adverse aortic event (rupture, dissection, death) for those with diabetes. However, the *p* -value of 0.520 was not statistically significant. While conclusions cannot be drawn, the identified trend may motivate further research on the impact of diabetes on TAA. Similarly, Kalogerakos et al 32 examined the impact of DM on natural history of ascending AAs. No clinically or statistically significant impact was found.

## Discussion

From a surgeon's viewpoint, a protective effect of diabetes, while initially counterintuitive, can make sense from an anatomical and mechanistic perspective. Experienced surgeons are fully aware that the diabetic aorta is thick and stiff—quite different from the usual connective tissue aneurysms that we encounter every day. This is why the diabetic aorta is known to hold sutures well.

To better understand the protective role of diabetes, we must recall Laplace's Law:



where T is wall tension, P is luminal pressure, r is aortic radius, and t is aortic wall thickness. Note that thickness resides in the denominator of the equation. In other words, a thicker aorta feels less wall tension and, thus, would naturally be expected to be protected. This fundamental principle of Laplace's Law may very well play a role in the observed diabetic protection of the aorta.

The general takeaway from the recent literature analyzing the relationship between diabetes and AAs supports a protective effect—with diabetes reducing growth rates and likelihood of rupture. However, gaps remain in terms of the mechanism of action and the impact on overall mortality. Further, studies continue to show a primary positive drug effect of both metformin and SGLT-2 inhibitors. This may motivate the use of these drugs for patients with AA, perhaps even without DM. The available studies, however, fail to analyze sociodemographic or environmental differences within this beneficial effect which could be noteworthy—for example, the potential impact of smoking cigarettes or the adequacy of blood pressure control or the closeness of medical follow-up. Additionally, research has been focused mainly on AAA, with relatively scant data on TAA.
